# Preparation of an Animal Suture

**Published:** 1891-12

**Authors:** D. A. Johnson


### PREPARATION OF AN ANIMAL SUTURE.

By D. A. Johnson, D. V. M.

It is a conceded fact that sutures made from animal tissue are preferable to those made of silk. After some experiments with silk, catgut, and other sutures, I conceived the idea of utilizing the tissues of the tendons of the horse for sutures. Taking one of the perforatus tendons from a mare that had died from a rupture of one of the iliac arteries in trying to foal, I placed it in a strong corrosive sublimate solution for one week; then I separated the tendon into fine threads, which I twisted by rolling on a clean board with the hand; then with a sharp knife removed the rough points caused by broken fibres. After having thus dressed the threads I placed them in a bottle containing olive oil three parts, and carbolic acid one part. The bottle was then tightly corked and set away for two weeks. Then the sutures were placed in another bottle containing olive oil and tannic acid—one drachm of the acid to the ounce of oil. This bottle was tightly corked and allowed to stand for two weeks; the sutures were then ready for use.

The corrosive sublimate and carbolic acid thoroughly disinfect the sutures, the tannic acid toughens them, and the olive oil keeps them soft and pliable. It is very difficult to get the threads as smooth as the catgut, and of late I have not attempted this, for when they are kept in the oil they are so pliable that they can be used conveniently if they are a little rough. Prepared as above delineated it takes four or five weeks for their absorption, in a closed cavity; or, as burried sutures. It is easier to tear the threads out of dried tendons, but they are more readily absorbed. These threads answer equally well for ligatures.

